# The *p250GAP* Gene Is Associated with Risk for Schizophrenia and Schizotypal Personality Traits

**DOI:** 10.1371/journal.pone.0035696

**Published:** 2012-04-18

**Authors:** Kazutaka Ohi, Ryota Hashimoto, Takanobu Nakazawa, Takeya Okada, Yuka Yasuda, Hidenaga Yamamori, Motoyuki Fukumoto, Satomi Umeda-Yano, Masao Iwase, Hiroaki Kazui, Tadashi Yamamoto, Masanobu Kano, Masatoshi Takeda

**Affiliations:** 1 Department of Psychiatry, Osaka University Graduate School of Medicine, Osaka, Japan; 2 Core Research for Evolutionary Science and Technology (CREST) of Japan Science and Technology Agency (JST), Saitama, Japan; 3 National Hospital Organization, Yamato Mental-Medical Center, Nara, Japan; 4 Molecular Research Center for Children's Mental Development, United Graduate School of Child Development, Osaka University, Kanazawa University and Hamamatsu University School of Medicine, Osaka, Japan; 5 Division of Oncology, Institute of Medical Science, University of Tokyo, Tokyo, Japan; 6 Department of Neurophysiology, Graduate School of Medicine, The University of Tokyo, Tokyo, Japan; 7 Department of Molecular Neuropsychiatry, Osaka University Graduate School of Medicine, Osaka, Japan; Chiba University Center for Forensic Mental Health, Japan

## Abstract

**Background:**

Hypofunction of the glutamate *N*-Methyl-d-aspartate (NMDA) receptor has been implicated in the pathophysiology of schizophrenia. p250GAP is a brain-enriched NMDA receptor-interacting RhoGAP. p250GAP is involved in spine morphology, and spine morphology has been shown to be altered in the post-mortem brains of patients with schizophrenia. Schizotypal personality disorder has a strong familial relationship with schizophrenia. Several susceptibility genes for schizophrenia have been related to schizotypal traits.

**Methods:**

We first investigated the association of eight linkage disequilibrium-tagging single-nucleotide polymorphisms (SNPs) that cover the *p250GAP* gene with schizophrenia in a Japanese sample of 431 schizophrenia patients and 572 controls. We then investigated the impact of the risk genetic variant in the *p250GAP* gene on schizotypal personality traits in 180 healthy subjects using the Schizotypal Personality Questionnaire.

**Results:**

We found a significant difference in genotype frequency between the patients and the controls in rs2298599 (*χ^2^* = 17.6, *p* = 0.00015). The minor A/A genotype frequency of rs2298599 was higher in the patients (18%) than in the controls (9%) (*χ^2^* = 15.5, *p* = 0.000083). Moreover, we found that subjects with the rs2298599 risk A/A genotype, compared with G allele carriers, had higher scores of schizotypal traits (*F_1,178_* = 4.08, *p* = 0.045), particularly the interpersonal factor (*F_1,178_* = 5.85, *p* = 0.017).

**Discussion:**

These results suggest that a genetic variation in the *p250GAP* gene might increase susceptibility not only for schizophrenia but also for schizotypal personality traits. We concluded that the *p250GAP* gene might be a new candidate gene for susceptibility to schizophrenia.

## Introduction

Schizophrenia is a common and complex psychiatric disease. The lifetime morbidity rate is 0.5–1.0% across distinct populations. Family, twin and adoption studies of schizophrenia have indicated that there are strong genetic factors and have estimated the rate of schizophrenia heritability at 80% [1], [2]. Although genes implicated in the pathogenesis of schizophrenia have been found using several approaches, such as through association studies of candidate genes, genome-wide association studies (GWAS), copy number variation (CNV) studies and pedigree studies [3], [4], the exact genetic factors of this complex disease remain to be explained.

Hypofunction of the glutamate *N*-Methyl-d-aspartate (NMDA) receptor is strongly implicated in the pathophysiology of schizophrenia. NMDA receptor antagonists, such as phencyclidine (PCP) and ketamine, mimic symptoms of the disorder in humans and exacerbate symptoms in patients with schizophrenia [5]. These NMDA receptor antagonists induce schizophrenia-like symptoms in humans. Preclinically, they have been shown to induce similar symptoms and to induce neural circuitry changes reminiscent of schizophrenia [6]. The ability of these NMDA receptor antagonists to induce a schizophrenia-like phenotype supports the concept that schizophrenia may be the result of reduced or abnormal functioning of NMDA receptors. Altered NMDA receptor binding density in several brain regions, such as in the anterior cingulate cortex, has been reported in schizophrenia [7], [8]. The NR2 subunits of the NMDA receptor are spatially and developmentally regulated, and they provide an important level of receptor regulation [9], [10]. NR2A and NR2B are the predominant subunits in the cortex, striatum and hippocampus [11], [12], [13]. In particular, these three areas are closely associated with the pathology of schizophrenia and with the neural circuits within and between these regions [14]. In patients with schizophrenia, alterations have been observed in the NR2 subunit mRNA and protein in the prefrontal cortex, including a reduction in NR2A mRNA and NR2B protein levels [15], [16]. Additionally, the NR2B subunit mRNA levels were increased in the hippocampus [17]. Therefore, different expression of NR2 subunits could play an important role in the pathophysiology of schizophrenia.

The NMDA receptor regulates activity-dependent spine morphological plasticity by modulating the actin cytoskeleton [18]. As the key regulators of actin cytoskeleton dynamics, the Rho family of GTPases, including RhoA, Cdc42, and Rac1 and their regulators, play an important role in NMDA receptor-mediated spine morphogenesis [18], [19]. In our previous study, we identified the *p250GAP* gene (also known as *p200RHOGAP*, *GRIT*, *KIAA0712*, *RICS*, or *ARHGAP32*: OMIM 608541) as a novel NMDA receptor-interacting RhoGAP [20], [21], [22], [23]. This gene spans approximately 56.17 kb of the genomic DNA and is located on chromosome 11q24.3. p250GAP is highly enriched in the central nervous system, is concentrated in the post-synaptic densities in neurons and is co-localized with the NR2B subunit of the NMDA receptor [20]. Knockdown of p250GAP increased spine width and elevated the endogenous RhoA activity in primary hippocampal neurons, suggesting that p250GAP regulates spine morphogenesis through its RhoGAP activity for RhoA [24]. Importantly, p250GAP activity and localization within neurons are regulated by NMDA receptor activity [20], [24], suggesting that p250GAP, together with the NMDA receptor, regulates NMDA receptor-mediated spine morphogenesis. Given that neuropathological studies of schizophrenia have shown alterations in spine morphology [25], [26], we hypothesized that the *p250GAP* gene may be related to the pathophysiology of schizophrenia. In this study, we investigated the association between the *p250GAP* gene and schizophrenia in a Japanese population using a gene-based approach.

Schizotypal personality disorder (SPD) is one of the schizophrenia spectrum disorders and is characterized by social avoidance, ideas of reference, vagueness, magical thinking, odd speech, illusions and paranoid ideation. The lifetime prevalence of SPD has been estimated at 3.9% [27], making it one of the more common psychiatric disorders. The prevalence rate of SPD in relatives of individuals with schizophrenia (6.9%) was higher than the prevalence rates found either in relatives of individuals with other psychiatric disorders or in mentally healthy subjects [28]. Twin studies have estimated that the heritability of the latent liability to SPD is 61–72% [29], [30]. Premorbid SPD is related to the development of schizophrenia [31]. These findings suggest that SPD shares common genetic influences with schizophrenia. The traits of SPD were incorporated in the SPD criteria in the *Diagnostic and Statistical Manual of Mental Disorders*, third edition (DSM-III), and the traits are listed in the DSM-IV-TR on Axis II. These traits can be identified using a well-validated questionnaire, such as the Schizotypal Personality Questionnaire (SPQ) [32]. The heritability rates of three schizotypal trait factors, cognitive/perceptual, interpersonal and disorganization, have been estimated at 40 to 60% [33], [34]. We recently demonstrated that a genome-wide genetic variant for schizophrenia in the *ZNF804A* gene was associated with schizotypal personality traits [35]. Additionally, we investigated whether a genetic variant in the *p250GAP* gene was associated with schizotypal personality traits in healthy subjects.

## Materials and Methods

### Ethics statement

Written informed consent was obtained from all subjects after the procedures had been fully explained. This study was performed in accordance with the World Medical Association's Declaration of Helsinki and approved by the Osaka University Research Ethics Committee.

### Subjects

The subjects of our genetic association study were 431 patients with schizophrenia (48.7% male (210 males, 221 females), mean age ± SD was 49.7±15.4 years) and 572 healthy controls (46.7% male (267 males, 305 females), mean age ± SD was 61.9±20.4 years). The sex ratio did not differ significantly between the groups (*χ^2^* = 0.41, *p* = 0.52), but the mean age was significantly different (*z* = −11.49, *p*<0.001). The subjects were all biologically unrelated and were Japanese. The subjects were recruited from both outpatient and inpatient units at Osaka University Hospital and other psychiatric hospitals. Each patient with schizophrenia had been diagnosed by at least two trained psychiatrists by unstructured clinical interviews, according to the criteria of the DSM-IV. When the diagnosis of the two trained psychiatrists was discordant, they discussed the diagnosis. When the diagnostic disputes were resolved and the patient was diagnosed as schizophrenic, we included the patient. When the diagnostic disputes were not resolved by discussion or the patient was not diagnosed as schizophrenia, we excluded the patient. Controls were recruited through local advertisements. Psychiatrically healthy controls were evaluated using unstructured interviews to exclude individuals with current or past contact with psychiatric services, with experience with psychiatric medications or who were not Japanese. We did not assess the controls for their family history of mental disorders, such as schizophrenia, bipolar disorder, or major depressive disorder. The ethnicity was determined by self-report and was not confirmed by genetic analyses.

Data for the schizotypal personality trait analysis were available for 180 healthy subjects [48.3% male (87/93), mean age ± SD: 36.5±11.5 years]. The subjects were included in the genetic association analysis. The subjects included in the analysis met additional criteria. Psychiatrically, medically and neurologically healthy controls were evaluated using the Structured Clinical Interview for DSM-IV-Non-Patient Edition (SCID-I/NP) to exclude individuals who had received psychiatric medications or who had first- or second-degree relatives with psychiatric disorders. Additionally, subjects were excluded from this study if they had neurological or medical conditions that could have potentially affected their central nervous system, such as atypical headaches, head trauma with loss of consciousness, chronic lung disease, kidney disease, chronic hepatic disease, thyroid disease, active cancer, cerebrovascular disease, epilepsy, seizures, substance-related disorders or mental retardation.

### SNP selection, genotyping and genomic sequencing

**Figure 1:**
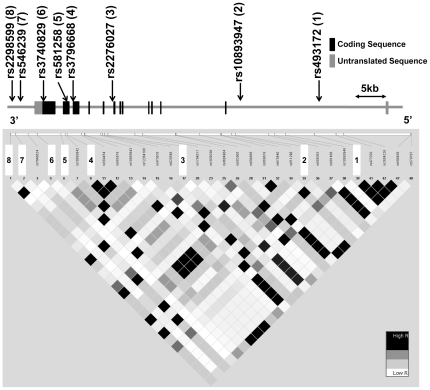
The genomic structure of *p250GAP* and linkage disequilibrium of the *p250GAP* in the HapMap JPT. The genomic structure of *p250GAP* is based on an entry in the Entrez Gene database (National Center for Biotechnology Information). The locations of the SNPs analyzed in this study are indicated by arrows. The numbers indicated in parentheses refer to the numbering of the SNPs in the linkage disequilibrium (LD) diagram. The distances of the exons-introns and the intermarkers are drawn to scale. The LDs between the pairwise SNPs are shown using the *r^2^* value at the bottom of the map of the gene structure for the HapMap JPT samples. High levels of LD are represented by black (*r^2^*) coloring, with increasing color intensity shown by the color bars.

This study was designed to examine the association between the *p250GAP* gene and schizophrenia by tagging single-nucleotide polymorphisms (SNPs) in the *p250GAP* gene and its flanking regions (±5 kb). Of the 31 SNPs in the *p250GAP* gene and flanking regions, we selected eight tagging SNPs using the TAGGER algorithm (Paul de Bakker, http://www.broad.mit.edu/mpg/tagger) with the criteria of *r^2^* greater than 0.5 in ‘pair-wise tagging only’ mode and a minor allele frequency (MAF) greater than 5%. The selection was implemented in Haploview 4.2 using HapMap data release 24/PhaseII Nov 08, on NCBI B36 assembly, dbSNP b126 (Japanese in Tokyo (JPT), Chr 11: 128,338,052..128,404,222) (Table S1). The eight tagging SNPs were rs493172, rs10893947, rs2276027, rs3796668, rs581258, rs3740829, rs546239 and rs2298599. The markers are shown in Table 1; the orientation and the alleles are reported on the genomic minus strand. The positions of the eight SNPs analyzed in the present study and the LD relationships between the SNPs in a HapMap JPT population are shown in Figure 1. Venous blood was collected from the subjects. Genomic DNA was extracted from the whole blood using standard procedures. The SNPs were genotyped using the TaqMan 5′-exonuclease allelic discrimination assay (Applied Biosystems; Foster City, California, USA) as previously described [36], [37]. Detailed information on the PCR conditions is available upon request. Genotyping call rates were 99.3% (rs493172), 98.9% (rs10893947), 99.1% (rs2276027), 99.7% (rs3796668), 98.4% (rs581258), 99.2% (rs3740829), 98.5% (rs546239) and 99.3% (rs2298599). No deviations from the Hardy-Weinberg equilibrium (HWE) in the examined SNPs were detected (*p*>0.05). Additionally, with 48 subjects with schizophrenia, we confirmed a SNP significantly associated with schizophrenia, genotyped by the TaqMan method, using direct DNA sequencing. These subjects were included in the genetic association analysis. The genomic regions were amplified by PCR using a pair of primers for rs2298599, 5′- AAGTCAGCCCAGACTCTCCA -3′ and 5′- GAGGGAGGAAGGGATTTTTG -3′. PCR for each sample was carried out in a total volume of 40 µl using a Gene Amp® PCR System 9700 (Applied Biosystems, CA, U.S.A). The PCR cycling conditions were 94°C for 10 minutes, 30 cycles at 94°C for 1 minute, 60°C for one minute and 72°C for 1 minute, followed by an incubation at 72°C for 10 minutes. The PCR products were purified using a QIA quick® PCR Purification Kit (QIAGEN, CA, USA), and the purification products were sequenced using a Big Dye® Terminator v1.1 Cycle Sequencing Kit (Applied Biosystems, CA, USA). Cycle sequencing conditions were 96°C for 2 minutes, 25 cycles of 96°C for 20 seconds, 50°C for 30 seconds and 60°C for 2 minutes, using a Gene Amp® PCR System 9700. The PCR products from the cycle sequencing were purified using a Big Dye® XTerminator™ Purification Kit (Applied Biosystems, CA, U.S.A.), and they were sequenced using an ABI PRISM® 3700 DNA Analyzer (Applied Biosystems, CA, U.S.A.). The sequencing was checked with SEQUENCHER ver. 4.7 (Gene Codes, U.S.A.).

**Table 1:** Genotypic and allelic distributions for SNPs in the *p250GAP* between patients with schizophrenia and controls.

Marker				SCZ (*n* = 431)			CON (*n* = 572)			Genotypic	SCZ	CON	Allelic	OR
SNP IDs	Position^a^	M/m	gene	M/M	M/m	m/m	M/M	M/m	m/m	*p* (*χ2*)	MAF		*p* (*χ2*)	(95% CI)
rs493172	128388089	C/G	intron1	346	77	3	451	116	3	0.63 (0.9)	0.10	0.11	0.49 (0.5)	0.90 (0.67–1.21)
rs10893947	128375634	G/A	intron1	122	217	88	177	294	94	0.25 (2.8)	0.46	0.43	0.14 (2.2)	1.15 (0.96–1.37)
rs2276027	128355514	T/C	intron8	241	158	27	303	229	36	0.57 (1.1)	0.25	0.27	0.42 (0.7)	0.92 (0.75–1.13)
rs3796668	128349062	A/C	intron11	186	182	62	206	292	72	**0.020 (7.8)**	0.36	0.38	0.22 (1.5)	0.89 (0.74–1.07)
rs581258	128348083	A/G	exon12	293	125	8	373	171	17	0.46 (1.6)	0.17	0.18	0.32 (1.0)	0.89 (0.70–1.12)
rs3740829	128344366	A/G	exon13	375	50	2	513	54	1	0.37 (2.0)	0.06	0.05	0.18 (1.8)	1.30 (0.89–1.91)
rs546239	128340968	A/G	3′	325	91	9	402	149	12	0.18 (3.4)	0.13	0.15	0.11 (2.6)	0.81 (0.63–1.05)
rs2298599	128340162	G/A	3′	167	184	76	219	296	53	**0.00015 (17.6)**	0.39	0.35	0.07 (3.3)	1.18 (0.99–1.42)

SCZ: patients with schizophrenia, CON: controls, M: major allele, m: minor allele, MAF: minor allele frequency, OR: odds ratio, 95%CI: 95% confidence interval.

a db SNP build 129.

All of the alleles are represented according to the minus strand DNA sequence. Numbers of genotypes were represented as genotype counts. *P* values<0.05 are in boldface and underlined.

### Schizotypal personality trait analysis

To assess schizotypal personality traits, a full Japanese version of the SPQ was administered to healthy subjects [38], [39]. The SPQ is a 74-item self-report questionnaire with a “yes/no” response format [40]. All items answered “yes” were scored 1. The SPQ measures nine subscales of specific schizotypal features, which are ideas of reference, odd beliefs/magical thinking, unusual perceptual experiences, suspiciousness/paranoid ideation, social anxiety, no close friends, constricted affect, eccentric/odd behavior and odd speech. The total SPQ score was obtained by summing the scores from all of the items. The three schizotypal trait factors, cognitive/perceptual, interpersonal and disorganization, were derived by summing the related subscale raw scores according to the three-factor model of Raine and colleagues [32]. Full-scale IQ was assessed using the Wechsler Adult Intelligence Scale, Revised or Third edition.

### Statistical analysis

Differences in clinical characteristics between the patients and the controls or between the genotype groups were analyzed using the *χ^2^* test for categorical variables and the Mann-Whitney *U*-test for continuous variables, using the PASW Statistics 18.0 software (SPSS Japan Inc., Tokyo, Japan). We performed power calculations using the Power Calculator for Two Stage Association Studies (http://www.sph.umich.edu/csg/abecasis/CaTS [41]). The power estimates were based on the allele frequency of 0.35 (rs2298599) in the controls and an alpha level of 0.05. Power was calculated under a prevalence of 0.01 using a multiplicative model that assumed varying degrees of the odds ratio (OR). Statistical analyses for the genetic associations were performed using the SNPAlyze V5.1.1 Pro software (DYNACOM, Yokohama, Japan). Deviation from the HWE was tested using *χ^2^* tests for goodness of fit. The allelic and genotypic distributions of *p250GAP* polymorphisms between the patients and the controls were analyzed using *χ^2^* tests.

Pairwise linkage disequilibrium (LD) analyses, expressed by *r^2^*, were applied to detect the intermarker relationships in each group using the Haploview 4.2 software (http://www.broad.mit.edu/mpg/haploview/contact.php). Haplotype frequencies were estimated using the maximum likelihood method with the genotyping data. We used the expectation-maximization algorithm from the SNPAlyze V5.1.1 Pro software. Rare haplotypes, detected in less than 3% of the patients and the controls, were excluded from the haplotypic association analysis, as previously described [42], [43]. Using a 2×2 contingency table approach, we performed 10,000 permutations of significance tests to determine empirical significance. We used a 2- to 8-window fashion analysis. We applied Bonferroni corrections in allelic and genotypic association analyses (eight tests) and in haplotypic association analyses (28 independent global tests).

The effects of the *p250GAP* genotype on the total score and on the three factors of the SPQ were analyzed by a one-way analysis of variance (ANOVA). To control confounding factors, the effect of the *p250GAP* genotype on the significance factor of the SPQ was analyzed by a one-way analysis of covariance (ANCOVA). Age, sex and education years were used as covariates because the SPQ total score and the three factors were correlated with these confounding factors in a previous study [44]. Standardized effect sizes were calculated using Cohen's *d* method (http://www.uccs.edu/faculty/lbecker). All *p* values are two tailed, and statistical significance was defined as *p*<0.05.

## Results

### Genetic association analysis

Our study size of 431 patients with schizophrenia and 572 controls had sufficient power (>80%) to detect a genetic effect at ORs of 1.30 or larger when the allele frequency was 0.35. The genotype and allele frequencies of the eight tagging SNPs located in the *p250GAP* gene and flanking regions are summarized in Table 1. We found significant differences in genotype frequencies between the patients and the controls in rs3796668 (*χ^2^* = 7.8, *p* = 0.020) and rs2298599 (*χ^2^* = 17.6, *p* = 0.00015). No allelic or genotypic associations were observed with schizophrenia for any other SNPs (*p*>0.05). The major genotype frequency of rs3796668 was significantly higher in the patients with schizophrenia (43%) than in the controls (36%) (*χ^2^* = 5.2, *p* = 0.023), but no differences were observed in the frequencies of the minor or heterozygous genotypes of rs3796668 (*p*>0.05). The minor genotype frequency of rs2298599 was higher in the patients with schizophrenia (18%) than in the controls (9%) (*χ^2^* = 15.5, *p* = 0.000083), but no differences were observed in the frequencies of the major or heterozygous genotypes of rs2298599 (*p*>0.05). The evidence for genotypic association of rs2298599 remained significant after a Bonferroni correction for multiple tests (corrected *p* = 0.0012). Genomic sequencing data for rs2298599 for each individual were in agreement with genotyping data using the TaqMan methods. Haplotype analysis showed a marginally significant association with schizophrenia in the rs3740829- rs546239- rs2298599 haplotype (*χ^2^* = 7.9, global *p* = 0.049) (Table S2). However, the association did not survive correction for multiple testing (*p*>0.05 after Bonferroni correction).

The LD relationships between the investigated markers are provided in Figure S1. The LD pattern observed in our controls was similar to our patients and the JPT HapMap samples; however, it was different from the LD pattern of the Utah residents with Northern and Western European ancestry from the CEPH collection (CEU) HapMap samples.

### *In silico* genotype-expression analysis

We examined an association between the rs2298599 and the expression levels of the *p250GAP* gene in immortalized lymphoblasts derived from 45 HapMap JPT subjects using WGAViewer software (http://compute1.lsrc.duke.edu/softwares/WGAViewer). However, *in silico* analysis revealed that there was no significant association between the SNP and the *p250GAP* expression in the immortalized lymphoblasts (*p* = 0.28).

### Impact of *p250GAP* genotype on schizotypal personality traits

**Figure 2:**
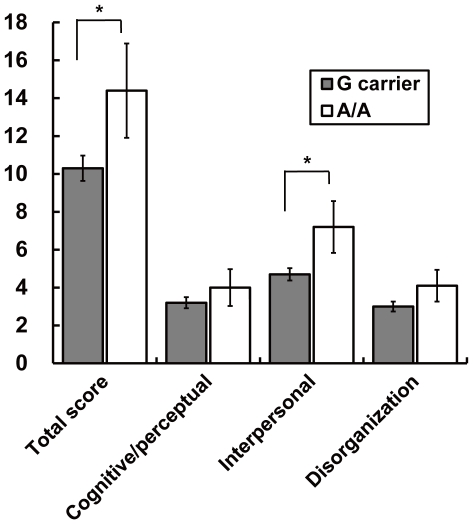
The association between the risk-associated *p250GAP* genotype and SPQ total score and the three factors. The gray bars represent individuals who are G-carriers (G/G and G/A genotypes) of rs2298599. The white bars represent individuals with the A/A genotype of the SNP. Error bars represent standard errors of the mean. * *p*<0.05.

We examined a possible association between the *p250GAP* genotype of rs2298599 and schizotypal personality traits in healthy subjects. Compared to controls, patients with schizophrenia were significantly more likely to carry the rs2298599 A/A genotype. Therefore, these analyses focused on a comparison of homozygous risk A/A genotype carriers versus homozygous carriers of one or two copies of the G allele (a combined G/G and G/A genotype group), under a recessive inheritance model of the risk A/A genotype. Demographic variables, age, sex, and full-scale IQ were not significantly different between the genotype groups, except for years of education (*z* = −2.05, *p* = 0.041) (Table 2). We first examined the possible effect of *p250GAP* rs2298599 on the total SPQ score and found a significant effect of the genotype (*F_1,178_* = 4.08, *p* = 0.045) (Table 3). Then, we investigated the genotype effects on the three SPQ factors, cognitive/perceptual, interpersonal and disorganization. A significant genotype effect was observed on the interpersonal factor (*F_1,178_* = 5.85, *p* = 0.017), but no significant genotype effects were observed on the cognitive/perceptual or disorganization factors (*p*>0.1). The effect of genotype on the interpersonal factor remained significant after adjusting for confounding factors (*F_1,175_* = 4.71, *p* = 0.031). Subjects with the risk A/A genotype of rs2298599 showed higher scores on schizotypal traits, particularly the interpersonal factor, compared with subjects with the G allele (Figure 2). The effect sizes of the total score and interpersonal factor were 0.41 and 0.47, respectively. When the two genotypes were divided into opposite two genotype groups (homozygous carriers of one or two copies of the A allele versus homozygous G/G genotype carriers) under a dominant model of inheritance, there was no significant difference in scores between A carriers and individuals with G/G genotype (*p*>0.05, Table S3).

**Table 2:** Demographic variables for subjects included in the SPQ analysis.

	Total	G carrier	A/A	*p* values	(*z*)
Variables	(*n* = 180)	(*n* = 159)	(*n* = 21)		
Age (years)	36.6±11.5	36.5±11.5	37.5±11.8	0.69	0.40
Sex (male/female)^a^	87/93	77/82	10/11	0.94	<0.01
Education (years)	15.4±2.4	15.6±2.4	14.4±2.0	**0.041**	−2.05
Full scale IQ	109.0±12.0	109.0±12.2	108.8±11.2	0.75	−0.33

Means ± SD are shown. *P* values<0.05 are in boldface and underlined.

a *χ^2^* test.

**Table 3:** Association of the *p250GAP* gene risk variant with the schizotypal personality traits.

SPQ	Total	G carrier	A/A		Genotype effect		
Variables	(*n* = 180)	(*n* = 159)	(*n* = 21)	Cohen's *d*	*F*_1,178_	*p* values	*η^2^*
Total score	10.7±8.9	10.3±8.4	14.4±11.4	−0.41	4.08	**0.045**	0.02
Cognitive/perceptual	3.3±3.8	3.2±3.7	4.0±4.5	−0.19	0.94	0.33	0.01
Interpersonal	5.0±4.5	4.7±4.1	7.2±6.3	−0.47	5.85	**0.017**	0.03
Disorganization	3.1±3.3	3.0±3.3	4.1±3.8	−0.31	2.13	0.15	0.01

SPQ: Schizotypal Personality Questionnaire. Means ± SD are shown. The effect sizes are typically categorized as small (*d* = 0.20, *η^2^* = 0.01), medium (*d* = 0.50, *η^2^* = 0.06) or large (*d* = 0.80, *η^2^* = 0.14). Significant *p* values are shown in boldface and underlined.

## Discussion

This study is the first investigation of the association of the *p250GAP* gene with schizophrenia. In this study, we first provided evidence that a genetic variant of the *p250GAP* gene was associated with the risk for schizophrenia. The frequency of individuals with the rs2298599 risk A/A genotype was higher in patients with schizophrenia than in the controls. Second, we indicated that the risk genotype of the *p250GAP* gene was associated with high schizotypal personality traits, particularly the interpersonal factor, in healthy subjects. Individuals with the rs2298599 risk A/A genotype scored higher on schizotypal personality traits and the interpersonal factor than did individuals with non-risk genotypes. These findings suggest that the *p250GAP* gene may be related to the risk for schizophrenia and the schizotypal personality traits.

Rs2298599 is situated within the relatively large LD block, which includes the *p250GAP* and the *P53AIP1* (OMIM 605426) genes (Figure S2). The SNP is located 2.9 kb downstream of the *p250GAP* gene and located 22.1 kb upstream of the *P53AIP1* gene. To confirm whether a significant association signal of rs2298599 with schizophrenia is attributed to the *p250GAP* gene, we checked strength of LDs in the genomic region (±50 kb) around rs2298599 using HapMap data (JPT, Chr 11: 128,290,162..128,390,161). Seven SNPs was related to rs2298599 with the criteria of *r^2^* greater than 0.8. Of the seven SNPs, five SNPs were located 5′ upstream from rs2298599 and four SNPs were included in the *p250GAP* gene. Two SNPs were located 5.2 and 7.9 kb downstream from rs2298599. These findings suggest that our association signal could be attributed to the *p250GAP* but not the *P53AIP1* gene. However, the *P53AIP1* may be a susceptibility gene for schizophrenia. Future studies are required to investigate the association between the *P53AIP1* and schizophrenia.

Although significant associations between the *p250GAP* gene and schizophrenia were observed in this study, no experimental evidence has indicated that the rs2298599 SNP of *p250GAP* is functional. To define a possibly functional SNP associated with the disease, followed by evaluation of altered function caused by the relevant SNP, may be able to narrow down the region of the association observed with the rs2298599. For example, gene expression analyses at either mRNA or protein levels of the *p250GAP* gene using postmortem or lymphoblast samples may be an alternative approach. We examined an association between the rs2298599 and *p250GAP* expression in immortalized lymphoblasts. However, *in silico* analysis revealed that the SNP might not be related to *p250GAP* mRNA expression in a Japanese population. A future biological study of the function of rs2298599 or *p250GAP* gene is required to verify our results.

MicroRNAs (miRs) regulate cellular fate by controlling the stability or translation of the mRNA transcripts. The miR132, located on 17p12.3, controls p250GAP protein levels and regulates neuronal morphogenesis by decreasing the levels of p250GAP [45]. The miR132 target sequence in the *p250GAP* 3′UTR, AACAGTCCACTGTCCAGCAGAGG, is conserved across vertebrate evolution. We performed a mutation search of the genomic region (the target sequence of miR132±250 bp) in the *p250GAP* gene using 48 patients with schizophrenia to evaluate the presence of a genetic variant in this region. However, we had no polymorphisms in our sequence data. This result suggests that the miR132 target sequence in the *p250GAP* might not play a major role in risk for schizophrenia.

Several molecular genetic studies have investigated the influences of susceptibility genes for schizophrenia on schizotypal personality traits. These studies have reported associations between the *COMT* [46], [47], [48], *NRG1* [49], *DTNBP1* [50], [51], *RGS4* [52], *DAAO* [51] and *ZNF804A* [35] genes and schizotypal components. Risk alleles or haplotypes of schizophrenia were correlated with high scores on schizotypal personality traits. Of these genes, the *COMT*, *NRG1*, *DTNBP1*, *DAAO* and *RGS4* genes, as well as the *p250GAP* gene, are directly or indirectly responsible for NMDA receptor-mediated glutamate transmission or signaling via glutamate receptors [53]. However, involvement of the glutamate NMDA receptors in SPD is still unknown. Further research will need to clarify the relationship between the glutamate NMDA receptors and SPD.

The interpretation of our results has several limitations. We found a significant association of the *p250GAP* gene with schizophrenia using 431 patients with schizophrenia and 572 controls. Our sample sizes had sufficient power (>0.80) to detect the effects of ORs of 1.30 or larger. Because our results were based on a relatively small sample to detect the effects of ORs of 1.30 or fewer, a future replication study using larger sample sizes is needed to confirm our findings. Our positive results might have been derived from a sample bias due to population stratification and non-age-matched samples, although the Japanese are a relatively homogeneous population. We used schizotypal personality traits as a phenotype of interest. As the assessment of the personality traits was based on a self-reported questionnaire, it was not an objective measurement. Importantly, to be included in the SPQ analysis, subjects were not required to meet criteria for SPD. We had hypothesized that schizotypal personality trait is a continuous measure of the genetic liability to schizophrenia. G allele carriers had marginally higher years of education and lower scores on schizotypal traits than did subjects with the risk A/A genotype. In a previous study, years of education had significant inverse effects on the total SPQ score and the three factor scores, indicating that the SPQ scores decreased with increased years of education [44]. The educational level difference between the genotype groups may have affected the genotype effects on the schizotypal personality trait. However, our results remained significant after adjusting for years of education.

In this study, we proposed *p250GAP* as a new candidate gene for susceptibility to schizophrenia. The association between the *p250GAP* gene and schizophrenia might partially explain the relationship between the hypofunction of the glutamate NMDA receptor and schizophrenia. Future studies are required to confirm the association between the *p250GAP* gene and schizophrenia in other populations.

## Supporting Information

Figure S1 **Linkage disequilibrium pattern of eight SNPs in the patient, control, HapMap JPT and CEU groups.** The linkage disequilibriums (LDs) between the pairwise SNPs are shown using the *r^2^* value separately for the patients with schizophrenia, the controls, the HapMap JPT samples and the HapMap CEU samples. High levels of LD (*r^2^*) are represented by black coloring, and increasing color intensity from 0 to 100 is shown by the color bars. The numbers (from 1 to 8) in the boxes refer to the eight tagging SNPs; rs493172 (1), rs10893947 (2), rs2276027 (3), rs3796668 (4), rs581258 (5), rs3740829 (6), rs546239 (7) and rs2298599 (8).
(TIF)

Figure S2 **Linkage disequilibrium in the genomic region (±50 kb) around rs2298599 SNP in HapMap JPT.** LD structure is based on an entry in the HapMap data release 24/PhaseII Nov 08, on NCBI B36 assembly, dbSNP b126 (JPT, Chr 11: 128,290,162..128,390,161). The LD structure between the pairwise SNPs is shown using the *r^2^* value. High levels of LD are represented by black (*r^2^*) coloring, with increasing color intensity.
(TIF)

Table S1 **Selected tagging SNPs in the** ***p250GAP*** **gene and its flanking regions.**
(DOC)

Table S2 **Haplotype analysis of the** ***p250GAP*** **gene between patients with schizophrenia and the controls.**
(DOC)

Table S3 **Association of the** ***p250GAP*** **gene variant with schizotypal personality traits under dominant model of inheritance.**
(DOC)
