# Supplementary figures and images for: The p250GAP Gene Is Associated with Risk for Schizophrenia and Schizotypal Personality Traits
Source: PLoS One. 2012 Apr 18;7(4):e35696. doi: 10.1371/journal.pone.0035696 (PMC3329470; doi:10.1371/journal.pone.0035696)

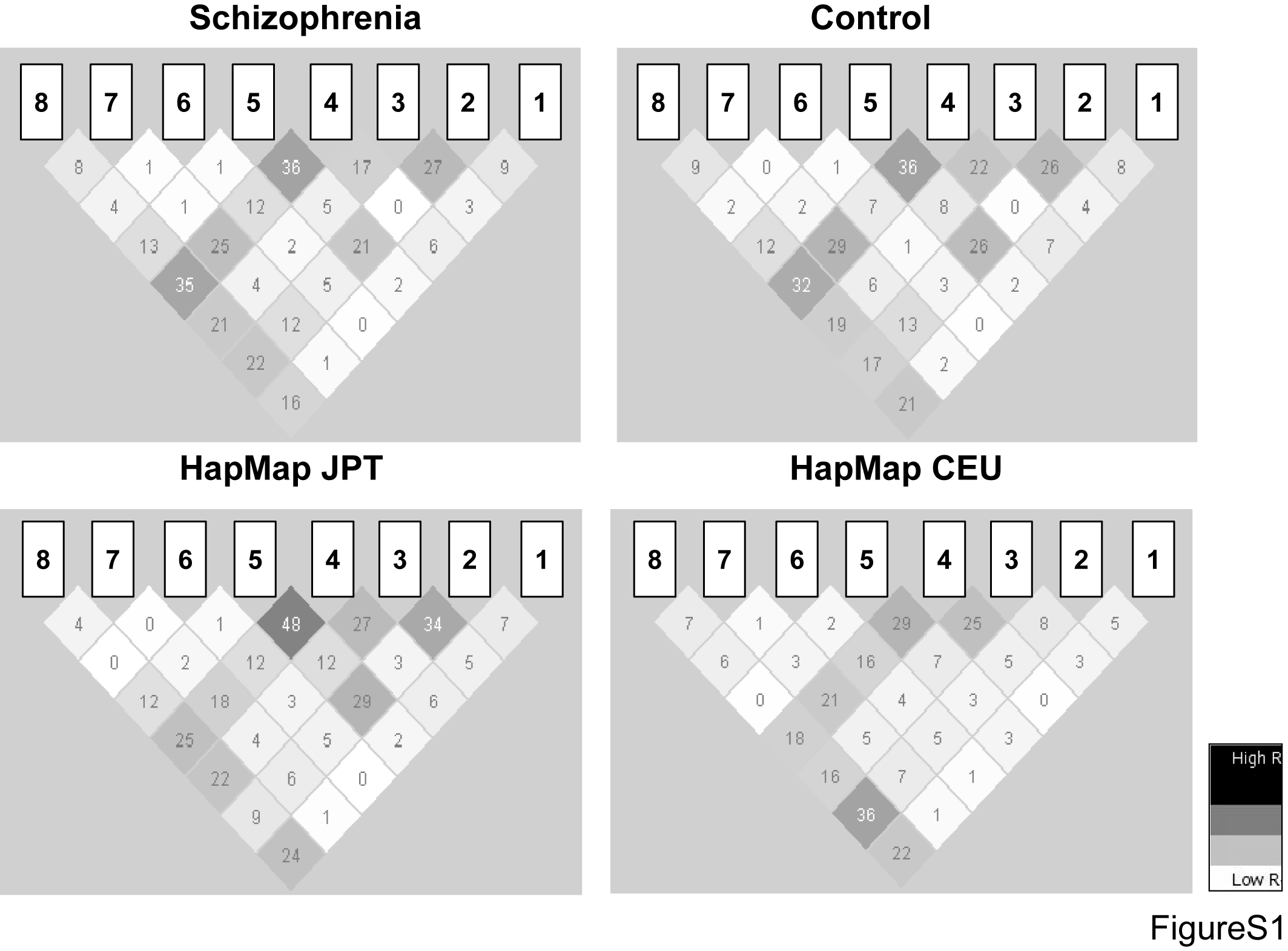

Supplement: Figure S1 — Linkage disequilibrium pattern of eight SNPs in the patient, control, HapMap JPT and CEU groups. The linkage disequilibriums (LDs) between the pairwise SNPs are shown using the r2 value separately for the patients with schizophrenia, the controls, the HapMap JPT samples and the HapMap CEU samples. High levels of LD (r2) are represented by black coloring, and increasing color intensity from 0 to 100 is shown by the color bars. The numbers (from 1 to 8) in the boxes refer to the eight tagging SNPs; rs493172 (1), rs10893947 (2), rs2276027 (3), rs3796668 (4), rs581258 (5), rs3740829 (6), rs546239 (7) and rs2298599 (8). (TIF) [file pone.0035696.s001.tif]

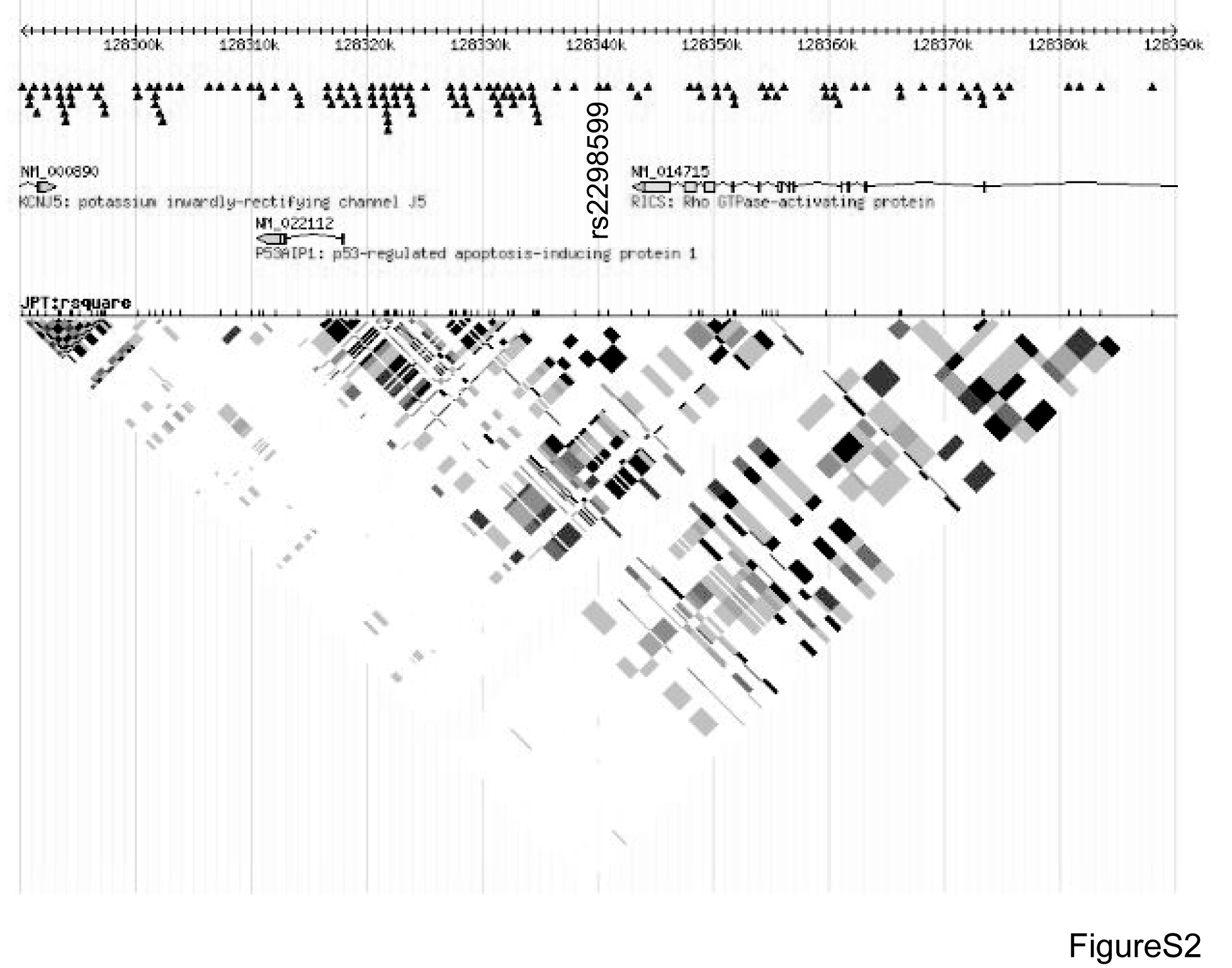

Supplement: Figure S2 — Linkage disequilibrium in the genomic region (±50 kb) around rs2298599 SNP in HapMap JPT. LD structure is based on an entry in the HapMap data release 24/PhaseII Nov 08, on NCBI B36 assembly, dbSNP b126 (JPT, Chr 11: 128,290,162..128,390,161). The LD structure between the pairwise SNPs is shown using the r2 value. High levels of LD are represented by black (r2) coloring, with increasing color intensity. (TIF) [file pone.0035696.s002.tif]
