# Supplementary material for: The p250GAP Gene Is Associated with Risk for Schizophrenia and Schizotypal Personality Traits
Source: PLoS One. 2012 Apr 18;7(4):e35696. doi: 10.1371/journal.pone.0035696 (PMC3329470; doi:10.1371/journal.pone.0035696)
Supplement: Table S1 — Selected tagging SNPs in the p250GAP gene and its flanking regions. (DOC) [file pone.0035696.s003.doc]

**Table S1 .** Selected tagging SNPs in the *p250GAP* gene and its flanking regions.

| Region numbers | SNPs included in each region | |  |  |  |  |  |  |
| --- | --- | --- | --- | --- | --- | --- | --- | --- |
| 1 | **rs493172** | rs2604226 | rs477070 | rs694668 | rs473616 | rs681408 | rs590458 | rs1784017 |
| 2 | **rs10893947** |  |  |  |  |  |  |  |
| 3 | **rs2276027** | rs10893943 | rs10893942 |  |  |  |  |  |
| 4 | **rs3796668** | rs579767 | rs670440 | rs665619 | rs564454 | rs669678 |  |  |
| 5 | **rs581258** | rs1939336 | rs12364106 |  |  |  |  |  |
| 6 | **rs3740829** |  |  |  |  |  |  |  |
| 7 | **rs546239** |  |  |  |  |  |  |  |
| 8 | **rs2298599** | rs511296 | rs10893948 | rs502905 | rs620548 | rs4604904 | rs588363 | rs7949524 |

The SNPs selected from each region are shown in boldface.
