# Supplementary material for: The p250GAP Gene Is Associated with Risk for Schizophrenia and Schizotypal Personality Traits
Source: PLoS One. 2012 Apr 18;7(4):e35696. doi: 10.1371/journal.pone.0035696 (PMC3329470; doi:10.1371/journal.pone.0035696)
Supplement: Table S2 — Haplotype analysis of the p250GAP gene between patients with schizophrenia and the controls. (DOC) [file pone.0035696.s004.doc]

**Table S2** Haplotype analysis of the *p250GAP* gene between the patients with schizophrenia and the controls.

|  |  |  | Haplotypic global *p* values (*χ2*) | | |  |  |
| --- | --- | --- | --- | --- | --- | --- | --- |
|  |  |  | Window level | |  |  |  |
| db SNP IDs | 2 | 3 | 4 | 5 | 6 | 7 | 8 |
| rs493172 |  |  |  |  |  |  |  |
|  | 0.30 (2.4) |  |  |  |  |  |  |
| rs10893947 |  | 0.41 (2.8) |  |  |  |  |  |
|  | 0.29 (2.5) |  | 0.18 (6.3) |  |  |  |  |
| rs2276027 |  | 0.24 (5.0) |  | 0.21 (6.0) |  |  |  |
|  | 0.16 (3.7) |  | 0.25 (5.4) |  | 0.07 (10.1) |  |  |
| rs3796668 |  | 0.27 (4.0) |  | 0.08 (9.8) |  | 0.08(10.1) |  |
|  | 0.46 (1.6) |  | 0.07 (8.5) |  | 0.08 (9.8) |  | 0.09 (9.7) |
| rs581258 |  | 0.13 (5.6) |  | 0.10 (7.9) |  | 0.08 (10.0) |  |
|  | 0.27 (2.7) |  | 0.21 (4.6) |  | 0.10 (7.9) |  |  |
| rs3740829 |  | 0.27 (4.0) |  | 0.13 (7.1) |  |  |  |
|  | 0.12 (4.2) |  | 0.15 (6.8) |  |  |  |  |
| rs546239 |  | **0.049 (7.9)** |  |  |  |  |  |
|  | 0.10 (4.7) |  |  |  |  |  |  |
| rs2298599 |  |  |  |  |  |  |  |

Haplotypes with frequencies < 3% in each group were excluded. Significant *p* value is shown in boldface and is underlined.
