# Supplementary material for: The p250GAP Gene Is Associated with Risk for Schizophrenia and Schizotypal Personality Traits
Source: PLoS One. 2012 Apr 18;7(4):e35696. doi: 10.1371/journal.pone.0035696 (PMC3329470; doi:10.1371/journal.pone.0035696)
Supplement: Table S3 — Association of the p250GAP gene variant with schizotypal personality traits under dominant model of inheritance. (DOC) [file pone.0035696.s005.doc]

**Table S3** Association of the *p250GAP* gene variant with schizotypal personality traits under dominant model of inheritance.

| **SPQ** | **Total** |  | **G/G** | **A carrier** |  |  | **Genotype effect** | | |
| --- | --- | --- | --- | --- | --- | --- | --- | --- | --- |
| **Variables** | **(*n* = 180)** |  | **(*n* = 66)** | **(*n* = 114)** | **Cohen's *d*** |  | ***F*_1,178_** | ***p* values** | ***η^2^*** |
| Total score | 10.7 ± 8.9 |  | 9.7 ± 8.3 | 11.3 ± 9.2 | -0.18 |  | 1.30 | 0.26 | 0.01 |
|  |  |  |  |  |  |  |  |  |  |
| Cognitive/perceptual | 3.3 ± 3.8 |  | 2.7 ± 3.6 | 3.6 ± 3.9 | -0.24 |  | 2.74 | 0.10 | 0.02 |
| Interpersonal | 5.0 ± 4.5 |  | 5.0 ± 4.1 | 5.1 ± 4.7 | -0.02 |  | <0.01 | 0.96 | <0.01 |
| Disorganization | 3.1 ± 3.3 |  | 2.7 ± 2.8 | 3.4 ± 3.6 | -0.22 |  | 1.74 | 0.19 | 0.01 |

SPQ: Schizotypal Personality Questionnaire. Means ± SD are shown. The effect sizes are typically categorized as small (*d*= 0.20, *η^2^*= 0.01), medium (*d*= 0.50, *η^2^*= 0.06) or large (*d*= 0.80, *η^2^*= 0.14).
